# Protocol for a systematic review of quantitative burn wound microbiology in the management of burns patients

**DOI:** 10.1186/s13643-015-0137-9

**Published:** 2015-11-06

**Authors:** Johnny Kwei, Fenella D. Halstead, Janine Dretzke, Beryl A. Oppenheim, Naiem S. Moiemen

**Affiliations:** Queen Elizabeth Hospital, University Hospitals Birmingham NHS Foundation Trust, Birmingham, England; Royal North Shore Hospital and Manly District Hospital, Northern Sydney Area Network, Sydney, NSW Australia; The Healing Foundation Burns Research Centre, Birmingham, England; NIHR Surgical Reconstruction and Microbiology Research Centre, Queen Elizabeth Hospital, Birmingham, England; Public Health, Epidemiology and Biostatistics, School of Health and Population Sciences, College of Medical and Dental Sciences, University of Birmingham, Edgbaston, Birmingham, England; Birmingham Children’s Hospital, Birmingham, England

**Keywords:** Burns, Quantitative microscopy, Quantitative microbiology, Infection, Sepsis, Mortality, Systematic review

## Abstract

**Background:**

Sepsis from burn injuries can result from colonisation of burn wounds, especially in large surface area burns. Reducing bacterial infection will reduce morbidity and mortality, and mortality for severe burns can be as high as 15 %.

There are various quantitative and semi-quantitative techniques to monitor bacterial load on wounds.

In the UK, burn wounds are typically monitored for the presence or absence of bacteria through the collection and culture of swabs, but no absolute count is obtained. Quantitative burn wound culture provides a measure of bacterial count and is gaining increased popularity in some countries. It is however more resource intensive, and evidence for its utility appears to be inconsistent.

This systematic review therefore aims to assess the evidence on the utility and reliability of different quantitative microbiology techniques in terms of diagnosing or predicting clinical outcomes.

**Methods/design:**

Standard systematic review methods aimed at minimising bias will be employed for study identification, selection and data extraction. Bibliographic databases and ongoing trial registers will be searched and conference abstracts screened. Studies will be eligible if they are prospective studies or systematic reviews of burn patients (any age) for whom quantitative microbiology has been performed, whether it is compared to another method. Quality assessment will be based on quality assessment tools for diagnostic and prognostic studies and tailored to the review as necessary. Synthesis is likely to be primarily narrative, but meta-analysis may be considered where clinical and methodological homogeneity exists.

**Discussion:**

Given the increasing use of quantitative methods, this is a timely systematic review, which will attempt to clarify the evidence base. As far as the authors are aware, it will be the first to address this topic.

**Trial registration:**

PROSPERO, CRD42015023903

## Background

Burn injuries are diverse but are unified in that they all involve liquefactive necrosis of the largest organ in the body, the skin. The skin is one of the most important immune defence mechanisms that the human body has. Consequently, infection is a significant problem in patients who survive an initial burn injury. This complication typically starts as bacterial colonisation and impacts significantly on morbidity, mortality and healthcare costs. Mortality for severe burns today stands at 5–15 % [1], with the majority of the mortality due to pneumonia (25 %), sepsis (26 %), urinary tract infections (22 %) and acute burn wound infections (5 %) [2].

Although there are a variety of infection routes which may lead to systemic infection and sepsis in the thermally injured patient (such as wound, intravenous access and chest infection), a key source of infection is the breached area of the skin. Bacteria can be introduced onto this surface in a number of different ways in the healthcare setting (via a number of exogenous and endogenous sources), leading to colonisation. This typically occurs in the form of a biofilm, a structured consortium of microbial cells surrounded by a self-produced polymer matrix. The longer the colonisation persists, the greater the likelihood of systemic infection. Furthermore, the greater the size of the breach, the greater the risks of bacterial invasion and systemic infection are thought to be.

Microbiological assessment can be with qualitative, semi-quantitative or quantitative methods. Assessment of burn wounds in the UK in general is qualitative and semi-quantitative, utilising swab cultures.

Various authors [3, 4] have suggested that qualitative and semi-quantitative methods should be replaced by fully quantitative bacteriology in order to improve patient management. The use of burn wound biopsies for histological (the staining and microscopic assessment of bacterial presence in different tissue planes) and quantitative (where an absolute quantity of bacteria per unit of volume is measured) microbiological assessment of the burn wound originates from the findings of Teplitz et al. [5], who found that in a rat model, increasing numbers of *Pseudomonas aeruginosa* on a burn wound were followed by invasion of the underlying viable tissue and clinical illness.

The clinical method for quantitative biopsy was described by Loebl et al. [6]; however, various authors [7, 8] since then have made modification to this method to better fit the parameters defined in their studies. Consequently, there now exist a variety of quantitative methods leading to an absolute bacterial count but no universally accepted ‘gold standard’. These methods differ in a number of ways, e.g. sample collection, method of biopsy and timing of collection.

The evidence for the utility of quantitative burn wound culture is inconsistent. Herruzo-Cabrera et al. [9] showed that a semi-quantitative surface swab method distinguished between wound contamination and infection, using a 10^5^ CFU/g threshold as the definition of infection by biopsy. This study was based on a porcine model where greater than 10^5^ surface bacterial counts were associated with tissue infection. Furthermore, an association between bacterial counts and wound healing has been suggested, with Heggers et al. [10] finding that when bacterial counts of 10^5^ CFU/g of tissue exist, wound healing is slowed (in an animal model) and Perry et al. [11] finding that skin graft take is adversely affected by high counts.

In contrast, Steer et al. [7] investigated the relationship between bacterial counts obtained by quantitative burn wound biopsy culture and quantitative surface alginate culture and clinical outcome following burn surgery and found that in patients with burns >15 % total body surface area (TBSA), a relationship between bacterial counts (obtained by either method) and subsequent sepsis or graft loss was not demonstrated.

The use of quantitative culture for the prediction of clinical outcomes is only one possible prognostic variable. Any evidence on the prognostic utility of bacterial count (whether as a single prognostic factor or in conjunction with others) needs to be evaluated in the context of the evidence on the accuracy and reliability of the counts obtained.

Given the increased use of quantitative methods in some burns centres, and the varied and sometimes conflicting evidence base, a systematic review of all existing evidence is warranted.

A scoping search was undertaken in MEDLINE, PubMed and the Cochrane library to identify existing (systematic) reviews and to gauge the volume of any primary studies on the topic. No existing systematic reviews were identified, but there are a number of primary studies, in addition to those mentioned above, exploring different aspects of quantitative microbiology, for example, related to measurement properties (e.g. inter-rater reliability, measurement errors), correlation with other culture methods or utility in predicting clinical outcomes such as sepsis.

A well-conducted systematic review is thus warranted in the use of quantitative microbiology in the management of burns patients.

## Methods/design

### Definitions

To the best of our knowledge, no standard definitions for qualitative or quantitative culture exist. For the purpose of the systematic review, the following will be used based on current literature [12]:

### General methods

This protocol has been guided by the PRISMA-P checklist [13]. The systematic review will be conducted using standard systematic review methodology based on the Cochrane handbook [14] and reported in accordance with the Preferred Reporting items for Systematic Reviews and Meta-Analyses (PRISMA) statement [13].

### Search strategy

A number of bibliographic databases will be searched: MEDLINE, Excerpta Medica Database (Embase), CINAHL, the Cochrane Database of Systematic Reviews, PubMed (the most recent 6 months), Cochrane Central Register of Controlled Trials (CENTRAL) and Scopus.

Publicly available trial registers (such as ClinicalTrials.Gov, UK Clinical Research Network Study Portfolio Database (UKCRN), WHO International Clinical Trials Registry Platform and the metaRegister of Controlled Trials (mRCT)) will be searched for all trials and the ZETOC database (British library) and Science Citation Index (Wed of Science) searched for conference proceedings.

In addition, abstracts from a number of national and international burns and microbiology conferences will be consulted from 2012 onwards in order to capture studies that are not yet fully published (by reviewing all posters and abstracts published in the meeting agenda). These include ANZBA (Australian New Zealand Burns Association), BBA (The British Burn Association), ABA (American Burn Association), EBA (European Burn Association), ISBI (International Society for Burn Injuries), EWMA (European Wound Management Association) and ECCMID (European Congress of Clinical Microbiology and Infectious Diseases). Citation checking of all included studies will also be undertaken.

The search strategy will use a combination of text word and MeSH terms relating to the population and the use of quantitative burn wound microbiology. Given that the research questions are broad, and aim to include any aspect of quantitative microbiology in burns patients, there will be no restriction by study design or outcomes.

There will be no restriction by publication date, language or publication type (e.g. abstract or full publication).

A sample search strategy for MEDLINE is shown. This will be adapted for use in other databases.

1. burn$.mp
2. exp burns/
3. “thermal injury”.mp
4. 1 or 2 or 3
5. biops$.mp
6. biopsy.mp
7. microscop*.mp
8. microscopy.mp
9. wound culture.mp
10. “quantitative adj3 culture”.mp
11. histology.mp
12. exp histology/
13. “bacterial count”.mp
14. swab$.mp
15. “microb* analy*”
16. “quantitative micro*”
17. 5 or 6 or 7 or 8 or 9 or 10 or 11 or 12 or 13 or 14 or 15 or 16
18. 4 and 17

### Selection criteria

The selection criteria are outlined below. All human studies relating to quantitative microbiology (biopsy culture and microscopy), and all possible clinical outcomes for burn patients will be included.

### Study design

The study design includes any prospective primary studies (excluding single case reports), or systematic reviews of such studies, assessing quantitative burn wound microbiology either on its own or compared to other methods.

### Types of participants

The participants are patients of any age (paediatric and adult) with burn injuries (of any size, mechanism or surface) for whom wound quantitative microbiology has been performed.

### Setting

Studies in any setting will be included.

### Intervention

This includes all methods of quantitative microbiology applied to diagnosis of burn wounds. Quantification will be as defined in Table 1.

**Table 1:** Definitions of qualitative, semi-quantitative and quantitative culture for wound swabs and biopsies

	Swab or biopsy
Qualitative	Presence or absence of growth
Semi-quantitative	Grading of the bacterial presence as either/or ± (scanty), + (few), ++ (moderate) and +++ (numerous) or as categories (e.g. <10^5^ CFU/g)
Quantitative	Where an absolute quantity is provided (following e.g. Miles and Misra quantification [22])

### Comparator

This includes either none or an alternative method (e.g. semi-quantitative or qualitative) of obtaining a bacterial count.

### Outcomes

- Measurement properties of different quantitative methods, e.g. in terms of reliability or repeatability
- Microbial load; quantitative (e.g. number of bacterial colony-forming units per gram of tissue (cfu/g) or per ml of liquid (cfu/ml)). Semi-quantitative studies will be included when used as a comparator in a study
- Diagnostic test accuracy measures (e.g. sensitivity or specificity)
- Clinical outcomes (e.g. colonisation, infection, antibiotic treatment, graft loss, bacteraemia, sepsis and mortality) and tolerance/acceptability
- Measures relating to the relationship between bacterial counts and clinical outcomes
- Resource related outcomes, e.g. length of hospital stay and cost

### Excluded

- Qualitative microbiological culture
- Semi-quantitative microbiological studies (unless used as a comparator with quantitative study)
- Animal studies
- Case reports

### Study selection

This will be a two-step process. Titles (and abstracts where available) will initially be screened by two reviewers, using pre-specified screening criteria.

Full texts of any potentially relevant articles will then be obtained, and the two reviewers will independently apply the full inclusion criteria. Any discrepancies between reviewers will be resolved by discussion or by referral to a third reviewer. Appropriate portions of non-English language articles will be translated where necessary and the study selection process documented using a PRISMA flow diagram [15]. Reference management software (Endnote) will be used to record reviewer decisions, including reasons for exclusion.

### Data extraction

All data will be extracted by two reviewers independently using a standardised, piloted data extraction form. Disagreements will be resolved through discussion or referral to third reviewer.

Data extraction will include (but not be limited to) the following variables:

- Study design and aim (e.g. from observational, prospective studies, randomised controlled trials and systematic reviews)
- Patient characteristics (e.g. paediatric and adult patients, severity of burn)
- Interventions given as part of standard care or for prevention/treatment of infection (e.g. topical/systemic antimicrobial regimens)
- Time and method of collection of the biopsy
- Method of quantitative microbiology (microscopy and culture) and any alternatives (if used)
- Outcomes (e.g. sepsis, mortality, presence/absence of infection, threshold of bacteria above or below 10^5^ cfu/g)
- Statistical analysis model utilised
- Effect sizes and measures of uncertainty
- Length of follow-up

Where sufficient information on results cannot be extracted, study authors will be asked to supply necessary information.

### Quality assessment

Quality assessment of included studies will be undertaken by two researchers independently, with any discrepancies resolved through discussion or referral to a third reviewer.

Items to be assessed will be based mainly on the Quality Assessment in Diagnostic Accuracy Studies (QUADAS)-2 criteria [15] but tailored to the review topic where appropriate. Criteria will relate to, for example, whether different methods for obtaining bacterial counts were performed independently from one another and with no knowledge of the results, whether all patients received/underwent testing with both methods and whether pre-specified thresholds were used for defining grade of infection.

It should be noted that quality assessment will be complicated by the fact that there is no recognised reference test (gold standard) and that studies may use different methods as their reference for defining levels of infection. Additional criteria will relate to (i) the sampling method, for example, whether the method for obtaining a sample for culture was performed in the same way for all patients and (ii) to the repeatability/reliability of the different methods where studies assessed this with duplicate samples or more than one assessor. Where studies report a prognostic element (i.e. where a bacterial count is linked to a later clinical outcome such as sepsis), relevant items from the Quality in Prognosis Studies (QUIPS) tool [16] will be used; these will include, for example, whether the outcome was clearly pre-defined in advance and whether confounding factors (such as antimicrobial treatment) were accounted for.

A sample of studies with different aims (e.g. looking at reliability of a method, comparing two or more methods or exploring a prognostic aspect) will be used to inform the final checklist in order to ensure that it can be used across different types of studies (providing they assess quantitative culture); however, it is likely that not all elements will be relevant for all included studies. Quality findings will be tabulated and synthesis will be narrative.

### Analysis/synthesis

The main outcome measure of the included studies is likely to be the absolute bacterial count per gram of tissue, either as a continuous variable or a dichotomous outcome (i.e. above or below one of more pre-defined thresholds). Bacterial counts may be compared between different methods, for example, with a correlation coefficient presented to show the strength of the relationship or a percentage above and below a certain threshold presented for different methods (or for duplicate samples). Studies may also present a positive or negative predictive value, i.e. chance of a person with a positive (negative) result obtained with one method actually having (not having) an infection (with the definition of infection based on another method). Sensitivities and specificities may also be presented, but interpretation is likely to be hampered by the lack of a validated reference standard.

Where studies have assessed the utility of bacterial counts in the prognosis of clinical outcomes (e.g. sepsis or graft loss), odd ratios or relative risks may be presented or calculable from raw data (i.e. patients above/below a certain threshold with/without the relevant clinical outcome).

Synthesis will most likely be narrative, with results for the same outcome metrics (e.g. correlation coefficient, percentage agreement and positive predictive value) tabulated and the direction of effect described, in the context of any clinical and methodological heterogeneity.

Heterogeneity is likely to be substantial, in the form of different methods for obtaining bacterial counts, different approaches to sampling (e.g. anatomical site, timing, number of samples), different laboratory approaches to culturing and different patient characteristics (e.g. severity of burn, co-morbidities). This will likely preclude meta-analysis, particularly for diagnostic test accuracy outcomes given the lack of a recognised reference test; formal evaluation of publication bias through funnel plots will therefore also not be possible. Visual presentation of results in forest or other plots without pooling may be explored should there be sufficient studies reporting the same outcome metric.

Potential sub-groups of interest may be based on studies using different quantitative microbiology methods, different semi-quantitative comparators, and different outcomes or with differences in patients’ antimicrobial therapy. The possibility of presenting findings for different sub-groups of studies will be explored; however, the anticipated scarcity of the overall evidence and the likely substantial heterogeneity may preclude this. For the same reasons, sensitivity analysis based on quality assessment is also unlikely to be feasible. However, the methodological quality of studies will be taken into account when drawing conclusions from findings.

## Discussion

Given the high mortality resulting from severe burn wound infection and sepsis, prevention of infection and/or early diagnosis of infection or sepsis are of utmost importance for patient care. In the UK, assessment of burn wounds is typically performed through the collection of charcoal swabs from the burnt surface, followed by standard culture on solid agar plates. This method results in a species identification and is either qualitative (presence or absence of bacteria is measured) or semi-quantitative (presence of bacteria is graded from scanty to heavy growth based on growth on a streaked agar plate) and is used to guide the duration of topical antimicrobial therapy.

This method is however limited in that it is difficult to differentiate between surface colonisation and deeper infection and, furthermore, provides little indication of the absolute number of bacteria present.

Since the advent of burn wound quantitative biopsy, there has been numerous studies evaluating its use, both in terms of its ability to reliably determine infection and to predict future adverse clinical outcomes (like graft loss or sepsis). As far as the authors are aware, there are no existing systematic reviews on this topic and the evidence from the modest volume of primary studies appears conflicting. There are also cost implications associated with quantitative microbiology, as this technique is more resource intensive than qualitative or semi-quantitative methods.

Various studies showed inconclusive efficacy. Loebl et al. [6] in 1974 introduced the use of quantitative microbiology in the assessment of burn wound patients and demonstrated that 88 % of paediatric and 45 % of adult burns patients with positive biopsies (>10^4^ organisms per gram of biopsy) developed clinical signs of sepsis and was a good predictor of burns sepsis. This predictive result of quantitative biopsy for sepsis was further supported by Buchanan et al. [17] in 1986.

However, McManus et al. [18] and Steer et al. [7], in the following decade, provided evidence that the positive results of quantitative biopsy did not correlate with depth of bacterial wound tissue invasion and sepsis. In fact, there is a suggestion that bacterial density from quantitative biopsies of the same wound at different sites varies significantly.

Globally, a large number of burn centres advocate and use a variety of microbiological methods (e.g. wound biopsy, swab cultures, capillary gauze, moist swab, absorbent disc, rapid slide and frozen section techniques) to quantify the bacterial bio-burden of a burn wound. Quantitative culture of a burn wound biopsy provides a measure of the bacterial count (in terms of colony-forming units (CFU)) per gram of tissue and is deemed to be superior than the non-quantitative methods, based on the presumption that there is a direct relationship between the bio-burden load on the surface to the risk of invasion, deeper infection, bacteraemia and sepsis and that certain thresholds of bacterial count can predict these possible clinical outcomes. 10^5^ CFU/g is commonly used as the threshold for distinguishing between colonisation and infection [3–5, 10, 11].

Quantitative cultures of wound biopsies are regularly used in the USA (in 47 % of centres) and Europe (in 27 % of centres) for diagnosis of burn wound infection; however, ≤5 % of burns centres [7] in the UK use this method. There is significant resources and time allocated to performing wound biopsies and cultures, as compared to standard wound swab and cultures.

Already, burn injuries pose a large financial strain, with healthcare costs in Australia ranging from AU$71,056 [19] (approximately £35,700) for a non-severe burns patient, to an average of greater than AU$500,000 [20] (approximately £251,000) for a severely burnt patient. Costs are similar in the USA, where the average cost for looking after a major burn patient is estimated to be US$200,000 (approximately £128,000), and more than US$18 billion is spent on specialised burn care annually [21]. No accurate financial data for the UK was identified, but costs are likely to be similarly high.

Despite this, quantitative microbiology is being increasingly used globally, particularly in the USA, and there is a growing interest in the UK as to whether this technique should be used more widely in order to provide the best possible care to burns patients.

The overall volume of evidence however is thought to be relatively sparse (<50 studies), and evidence for the practice is unclear. The proposed systematic review therefore aims to review all evidence relating to any aspect of quantitative culture and to highlight any gaps in the evidence base.

There is anticipated heterogeneity in the results of this systematic review. Preliminary search also showed variability in study design, with the use of quantitative microbiology in comparison to histology, bacteraemia, sepsis and mortality in an inconsistent manner.

Specifically, any evidence relating to the following questions will be reviewed:

i) Measurement properties of quantitative burn wound biopsy culture (with histological microscopy), in terms of internal consistency, reliability and measurement error
ii) Measurement properties of quantitative burn wound biopsy culture (with histological microscopy) compared to other methods of quantification (swab culture or other methods)
iii) Correlation between bacterial wound density and colonisation, bacterial invasion and/or systemic infection (e.g. bacteraemia and sepsis)
iv) Correlation between bacterial wound counts and likelihood of progression to bacteraemia and sepsis (i.e. prognostic value of bacterial counts obtained by quantitative microbiology)
v) Any models evaluating multiple prognostic factors (including bacterial counts obtained by quantitative microbiology) in terms of progression to bacterial invasion, bacteraemia or sepsis.

Diagnostic accuracy is unlikely to have been evaluated in the absence of a gold standard for obtaining a bacterial count; however, studies measuring this will also be included.

A systematic review, which synthesises all the available evidence, is therefore urgently required. An evaluation of the utility of the bacterial count for predicting clinical outcomes may be hampered by a lack of evidence (and vast heterogeneity) on the validity of any one method for obtaining a count; however, any gaps in the evidence and/or uncertainties around findings will be highlighted. An overall appraisal of the study methodology and risk of bias of all included studies will help to make an assessment of the robustness of findings and may also help to inform the study design of any future research.

In order to provide the best standard of care for patients in the UK and justify the cost effectiveness of quantitative microbiology, a well-conducted systematic review will define the deficiencies in evidence-based practice and will hopefully be identified as a recommendation for clinical practice in the current treatment of burn patients.

If areas of deficiency in evidence for practice are identified, this may instigate future research in these areas of quantitative microbiology.

## Abbreviations

ABA: American Burn Association
ANZBA: Australian and New Zealand Burns association
BBA: The British Burn Association
EBA: European Burn Association
ECCMID: European Congress of Clinical Microbiology and Infectious Diseases
EWMA: European Wound Management Association
ISBI: International Society for Burn Injuries
PRISMA: Preferred Reporting Items for Systematic Reviews and Meta-analyses
QUADAS: Quality Assessment of Diagnostic Accuracy Studies
QUIPS: Quality Model of Investigating Prognostic Studies

## References

[1] Church D, Elsayed S, Reid W, Winston B, Lindsay R (2006). Burn wound infections. Clin Micro Rev.

[2] Sheppard NN, Hemington-Gorse S, Shelley OP, Philp B, Dziewulski P (2011). Prognostic scoring systems in burns: a review. Burns.

[3] Krizek TJ, Robson MC, Kho E (1967). Bacterial growth and skin graft survival. Surg Forum.

[4] Robson MC, Shaw RC, Heggers JP (1968). Quantitative bacteriology and delayed wound closure. Surg Forum.

[5] Teplitz C, Davis D, Mason AD, Moncrief JA (1964). Pseudomonas burn wound sepsis. I: Pathogenesis of experimental pseudomonas burn wound sepsis. J of Surg Res.

[6] Loebl EC, Marvin JA, Heck EL, Curreri W, Baxter CR (1974). The use of quantitative biopsy cultures in bacteriologic monitoring of burn patients. J Surg Resea.

[7] Steer JA, Papini RPG, Wilson APR, McGrouther DA, Parkhouse N (1996). Quantitative microbiology in the management of burn patients. II. Relationship between bacterial counts obtained by burn wound biopsy culture and surface alginate swab culture, with clinical outcome following burn surgery and change of dressings. Burns.

[8] Uppal SK, Ram S, Kwatra B, Garg S, Gupta R (2007). Comparative evaluation of surface swab and quantitative full thickness wound biopsy culture in burn patients. Burns.

[9] Herruzo-Cabrera R, Vizcaineo-Alcaide MJ, Pinedo-Castilllo C, Rey-Calero J (1992). Diagnosis of local infection of a burn by semiquantitative culture of the eschar surface. J Burn Care Rehabil.

[10] Heggers JP, Haydon S, Ko F, Hayward PG, Carp S, Robson MC (1992). Pseudomonas aeruginosa exotoxin A: its role in retardation of wound healing: the 1992 Lindberg award. J Burn Care & Rehab.

[11] Perry AW, Howard SS, Lawrence JG, Wayne KS, Thomas JK (1989). Skin graft survival—the bacterial answer. Ann of Plast Surg.

[12] Kallstrom G (2014). Are quantitative bacterial wound cultures useful?. J of Clin Micro.

[13] Moher D, Liberati A, Tetzlaff J, Altman DG, Group P (2009). Preferred reporting items for systematic reviews and meta-analyses: the PRISMA statement. BMJ (clinical research edition).

[14] Higgins JPT, Green S (editors). *Cochrane Handbook for Systematic Reviews of Interventions* Cochrane Handbook for Systematic Reviews of Interventions and meta-an Available from www.cochrane-handbook.org.

[15] Whiting PF, Rutjes AWS, Westwood ME (2011). and the QUADAS-2 group. QUADAS-2: a revised tool for the quality assessment of diagnostic accuracy studies. Ann Intern Med.

[16] Croll PR, Croll J (2004). QUIPS UA quality model for investigating risk exposure in e-health systems. Stud Health Technol Inform.

[17] Buchanan K, Heimbach DM, Minshew BH, Coyle MB (1986). Comparison of quantitative and semiquantitative culture techniques for burn biopsy. J Clin Micro.

[18] McManus AT, Kim SH, McManus WF, Mason AD, Pruitt BA (1987). Comparison of quantitative microbiology and histopathology in divided burn-wound biopsy specimens. Arch Surg.

[19] Ahn CS, Maitz PK (2012). The true cost of burn. Burns.

[20] Greenwood JE (2009). Burn injury and explosions: an Australian perspective. Open Access J of Plast Surg.

[21] Pruitt BA, McManus AT, Kim SH, Goodwin CW (1998). Burn wound infections: current status. World J Surg.

[22] Miles AA, Misra SS, Irwin JO (1938). The estimation of the bactericidal power of the blood. J Hygiene.

